# Accuracy of a novel real-time continuous glucose monitoring system: a prospective self-controlled study in thirty hospitalized patients with type 2 diabetes

**DOI:** 10.3389/fendo.2024.1374496

**Published:** 2024-05-21

**Authors:** Shenghui Ge, Hui Zhang, Jun Wang, Huiqin Li, Xiaofei Su, Dafa Ding, Jianhua Ma

**Affiliations:** ^1^ Department of Endocrinology, Nanjing First Hospital, Nanjing Medical University, Nanjing, China; ^2^ Department of Endocrinology, The Second Affiliated Hospital of Nanjing Medical University, Nanjing, China; ^3^ Department of Health Management Center, Nanjing First Hospital, Nanjing Medical University, Nanjing, China; ^4^ Department of Endocrinology, The Second Affiliated Hospital of Nanjing Medical University, Nanjing, China

**Keywords:** Glunovo^®^, rtCGMS, type 2 diabetes, flash glucose monitoring, venous blood glucose

## Abstract

**Aims:**

The present study aimed to investigate the accuracy of the Glunovo^®^ real-time continuous glucose monitoring system (rtCGMS).

**Methods:**

We conducted a 14-day interstitial glucose level monitoring using Glunovo^®^ rtCGMS on thirty hospitalized patients with type 2 diabetes. The flash glucose monitoring (FGM) was used as a self-control. Consistency tests, error grid analysis, and calculation of the mean absolute relative difference (MARD) were performed using R software to assess the accuracy of Glunovo^®^ rtCGMS.

**Results:**

Glunovo^®^ exhibited an overall MARD value of 8.89% during hospitalization, compared to 10.42% for FGM. The overall percentages of glucose values within ±10%/10, ± 15%/15, ± 20%/20, ± 30%/30, and ±40%/40 of the venous blood glucose reference value were 63.34%, 81.31%, 90.50%, 97.29%, and 99.36% for Glunovo^®^, respectively, compared with 61.58%, 79.63%, 88.31%, 96.22% and 99.23% for FGM. The Clarke Error Grid Analysis showed that 99.61% of Glunovo^®^ glucose pairs and 100.00% of FGM glucose pairs within zones A and B.

**Conclusion:**

Our study confirms the superior accuracy of Glunovo^®^ in monitoring blood glucose levels among hospitalized patients with type 2 diabetes.

## Introduction

Diabetes and its complications impose a heavy burden on patients. It is estimated that the global diabetes prevalence among individuals aged 20–79 will increase to 12.2% (783.2 million) (1). Effective management of blood glucose levels is paramount for individuals with diabetes, as abnormal levels can cause irreversible damage to the cardiovascular and nervous systems (2, 3). Traditional self-monitoring of blood glucose (SMBG) often poses challenges due to its painful and inconvenient nature, hindering standardized blood glucose management. Moreover, while HbA1c provides an average of long-term blood glucose levels, it fails to capture short-term fluctuations (4). Continuous glucose monitoring systems (CGMS) have emerged as a solution to address these limitations. CGM measures glucose concentration in the interstitial fluid rather than blood, and its values are determined by the rate of glucose diffusion from plasma to interstitial fluid and the rate at which subcutaneous tissue cells take up glucose (5). Currently, two types of CGMS are available: flash glucose monitoring (FGM) or intermittently scanned CGMS (isCGMS), and real-time CGMS (rtCGMS) (6).

Glunovo^®^ is an rtCGMS consisting of a sensor, transmitter, and a mobile application for data analysis. The sensor, designed for subcutaneous installation, has a 14-day lifespan. It generates electrical signals, which are transmitted to the mobile application for display of blood glucose readings. While previous studies have indicated the stability and repeatability of Glunovo^®^, there remains a lack of head-to-head research to evaluate its accuracy (7). To address this gap, we conducted a head-to-head study to assess the accuracy of Glunovo^®^.

## Methods

### Study design and study population

Patients with type 2 diabetes who underwent standardized treatment at the Nanjing First Hospital from March 2019 to October 2019 were enrolled in this study.

### Inclusion criteria

(1) Age: 18–70 years.
(2) Confirmed diagnosis of type 2 diabetes with a duration of at least 3 months.
(3) No participation in other clinical studies in the past 3 months.

### Exclusion criteria

(1) Pregnancy or breastfeeding.
(2) History of adhesive tape allergy.
(3) Acute diabetes complications (e.g., diabetic ketoacidosis and hyperglycemic hyperosmolar coma).
(4) Severe immunosuppressive disorders or systemic neurological diseases.

### Data collection

(1) General clinical data, including name, age, gender, systolic blood pressure (SBP), diastolic blood pressure (DBP), body mass index (BMI), hemoglobin A1c (HbA1c), triglyceride (TG), creatinine and duration of diabetes.
(2) Blood glucose values recorded from two groups of CGM devices at three stages: initial (1st or 2nd day), intermediate (7 ± 1 days), and final (14th day), along with paired venous blood glucose measurements.

### Details of Glunovo^®^

The Glunovo^®^ device featured a 14-day real-time glucose oxidase electrochemical sensor with a flexible sensor probe. Glucose and oxygen from tissue fluid permeate the probe, triggering an electrochemical reaction that generates an electrical signal. This signal, emitted every 3 minutes, was processed by a transmitter (7 mm thick, with a lifespan of 3 years), an applicator for the transmitter applied by a simple click, and software for processing and sharing data. The applicator, designed for ease of use, included a button to position the sensor and retract the insertion needle upon pressing.

The processed signals from the transmitter were converted into blood glucose readings, transmitted via Bluetooth to a mobile application. The application provided real-time display of blood glucose readings, reflected glucose fluctuation trends through trend curves, and enabled exportation of historical data. The analysis software could analyze exported data from the application and conduct statistical analyses for a deeper understanding of the titration of anti-diabetic drugs. All sensors were clinically implanted using an automatic abdominal sensor applicator, with each participant receiving two sensors for improved performance. Paired sensors values were calculated using pairwise average absolute difference and matched to corresponding venous blood glucose levels. In case of sensor failure, the replacement sensor would match the venous blood glucose value.

### Procedures

All participants underwent a 14-day adaptation period using the CGMS. Following the sensor’s recommendations, the device calibrated twice daily using SMBG measurements every 24 hours. After the 14-day adaptation period, paired continuous glucose values and venous blood glucose values were collected for each participant, with a minimum of 24 readings collected within different time periods over 7 hours. The collection of paired continuous glucose and venous blood glucose readings was randomized, assigning each participant a random collection period divided into three stages: initial, middle, and final. FGM was performed as a matched control during this period.

Real-time blood glucose values measured by Glunovo^®^ were compared with venous blood glucose values measured by hospital nurses using the EKF Fast Blood Glucose Analyzer (Biosen-C-Line, EKF Diagnostics, Cardiff, UK). The measurement range of Glunovo^®^ was approximately 2.2–22.2 mmol/L; values outside this range were not included in the analysis. The study was conducted in accordance with the Helsinki Declaration of 1964 and its subsequent amendments and received ethical approval from the Ethics Committee of Nanjing First Hospital (Approval Number: ChiCTR2100045233).

### Statistical analysis

For continuous variables, Shapiro-Wilk test was used to assess normality. Normally distributed data were presented as mean ± SD, and non-normally distributed data as median (interquartile range). Categorical variables were presented as count (percentage). Mean absolute relative difference (MARD) was determined as the average relative difference between the CGMS and venous blood glucose pairs and expressed as a percentage. CGM performance evaluation followed statistical recommendations from Clarke and Kovatchev (8). The numbers of glucose pairs in various risk zones of error grid analyses were determined with the R package “ega,” which is designed for Clarke or Parkes error grid analysis (https://cran.r-project.org/web/packages/ega/ega.pdf). A p-value less than 0.05 was considered statistically significant. All statistical calculations were performed using R software (version 4.3.1).

## Results

### Baseline characteristics and venous blood glucose

A total of 31 patients were enrolled, with one participant dropping out midway, resulting in the final collection of data from 30 patients. The patients’ characteristics were presented in **Table 1**, including 18 females and 12 males, with a median age of 56.00 years and an average BMI of 24.55 kg/m^2^. The median duration of diabetes was 9.00 years, with average SBP and DBP of 123.60 mmHg and 75.07 mmHg, respectively. Blood indicators, including HbA1c, TG, and creatinine, were 7.81%, 1.41 mmol/L, and 64.31 μmol/L, respectively. A total of 2,327 pairs of matched glucose data were available for evaluation. Venous blood glucose levels were categorized as <3.9 mmol/L (6 pairs), 3.9–10.0 mmol/L (1,422 pairs), and ≥10.0 mmol/L (899 pairs).

**Table 1:** Baseline patient characteristics.

Subject	Data
Total	30
Gender (N, %)	
Male	12 (40.00%)
Female	18 (60.00%)
Age (years)	56.00 (51.25 - 61.00)
BMI (kg/m^2^)	24.55 ± 2.78
HbA1c (%)	7.81 ± 1.52
SBP (mmHg)	123.60 ± 13.97
DBP (mmHg)	75.07 ± 9.74
TG (mmol/L)	1.41 (0.89 - 2.09)
Creatinine (μmol/L)	64.31 ± 12.42
Duration of diabetes (years)	9.00 (6.25 - 12.00)

BMI, body mass index; HbA1c, glycosylated Hemoglobin; SBP, systolic blood pressure; DBP, diastolic blood pressure; TG, triglyceride.

### CGM performance and error grid analysis

MARD values were shown in **Table 2**. Overall, the MARD for Glunovo^®^ was 8.89%, and for FGM, it was 10.42%. The data were further categorized into rate of change in venous blood glucose groups defined by intervals: <-0.11, (-0.11, -0.06], (-0.06, 0], (0, 0.06], (0.06, 0.11], >0.11 mmol/L/min. The Glunovo^®^ exhibited MARD values of 10.09%, 7.44%, 7.93%, 9.41%, 12.70%, and 17.11% for these respective intervals, whereas the FGM demonstrated MARD values of 10.73%, 9.81%, 10.12%, 10.19%, 11.25%, and 21.30%. For venous blood glucose categorizations: < 3.90, [3.90, 10.00), ≥ 10.00 mmol/L, Glunovo^®^ exhibited MARD values of 8.65%, 8.09%, and 10.58%, respectively, while FGM demonstrates MARD values of 15.21%, 9.60%, and 8.57%. In the initial, middle, and final stages of data collection, MARD values were 8.65%, 8.09%, and 10.58% for Glunovo^®^, while 15.21%, 9.60%, and 8.57% for FGM.

**Table 2:** Comparison of MARD values between Glunovo^®^ and FGM.

Group	FGM (%)	Glunovo^®^ (%)
Total	10.42	8.89
Venous blood glucose (mmol/L)		
< 3.90*	13.12	25.16
[3.90, 10.00)	11.53	7.93
≥ 10.00	8.64	10.29
ROC (mmol/L/min)		
< -0.11	10.73	10.09
[-0.11, -0.06)	9.81	7.44
[-0.06, 0)	10.12	7.93
[0, 0.06)	10.19	9.41
[0.06, 0.11)	11.25	12.70
≥ 0.11	21.30	17.11
Period of data collection		
Initial stage	10.59	8.83
Intermediate stage	7.51	9.03
Final stage	13.03	8.90

*There were only six paired matched glucose values for glucose readings 3.90 mmol/L. MARD, mean absolute relative difference; FGM, flash glucose monitoring; ROC, rate of change in venous blood glucose.

Agreement analyses were presented in **Table 3**. The overall percentages of glucose values within ±10%/10 mmol/L, ± 15%/15 mmol/L, ± 20%/20 mmol/L, ± 30%/30 mmol/L, and ±40%/40 mmol/L of the venous blood glucose reference value were 63.34%, 81.31%, 90.50%, 97.29%, and 99.36% for Glunovo^®^, respectively, compared with 61.58%, 79.63%, 88.31%, 96.22% and 99.23% for FGM.

**Table 3:** Agreement analysis between Glunovo^®^ and FGM.

Category	± 10/10%	± 15/15%	± 20/20%	± 30/30%	± 40/40%
FGM	61.58	79.63	88.31	96.22	99.23
(95% CI)	(61.56, 61.60)	(79.61, 79.65)	(88.3, 88.32)	(96.21, 96.23)	(99.22, 99.23)
Glunovo^®^	63.34	81.31	90.50	97.29	99.36
(95% CI)	(63.32, 63.36)	(81.29, 81.32)	(90.49, 90.51)	(97.29, 97.30)	(99.35, 99.36)

FGM, flash glucose monitoring.

**Figure 1:**
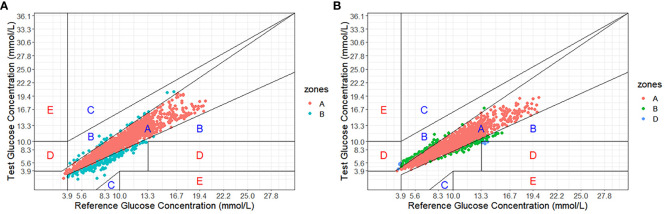
Clarke error grid analysis. **(A)** flash glucose monitoring; **(B)** Glunovo^®^. The percentage of measuring points falling in A + B zones was 100.00% for flash glucose monitoring and 99.61% for Glunovo^®^. Zone A, clinically accurate; Zone B, benign errors; Zone C, overcorrection errors; Zone D, failure to treat errors; and Zone E, erroneous treatment errors.

**Figure 2:**
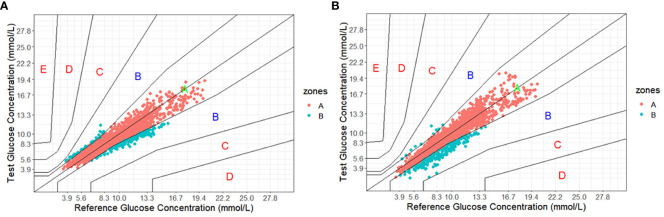
Parkes error grid analysis. **(A)** flash glucose monitoring; **(B)** Glunovo^®^. The percentage of measuring points falling in A + B zones was 100.00% for flash glucose monitoring and 100.00% for Glunovo^®^. Zone A, clinically accurate; Zone B, benign errors; Zone C, overcorrection errors; Zone D, failure to treat errors; and Zone E, erroneous treatment errors.

As shown in **Figure 1**, Clarke Error Grid Analysis demonstrated acceptable clinical accuracy. For Glunovo^®^, 99.61% of glucose values fell within zones A (93.64%, n = 2,179) and B (5.97%, n = 139). In comparison, for FGM, 100.0% of glucose values were within zones A (90.29%, n = 2,101) and B (9.71%, n = 226). As shown in **Figure 2**, Parkes Error Grid Analysis demonstrated acceptable clinical accuracy. For Glunovo^®^, 100.0% of glucose values fell within zones A (92.52%, n = 2,153) and B (7.48%, n = 174). In comparison, for FGM, 100.0% of glucose values were within zones A (90.29%, n = 2,101) and B (9.71%, n = 226).

## Discussion

Our findings demonstrated that the Glunovo^®^ exhibited high accuracy, with an overall MARD of 8.89%. In the initial, middle, and final stages of data collection, Glunovo^®^ consistently exhibited excellent performance. The 2013 CGM Roundtable emphasized that MARD values below 14% are desirable, while values exceeding 18% indicate poor accuracy (9). In comparison, the FGM system exhibited a slightly higher MARD value of 10.42%. A study of 72 diabetic patients evaluated a Dexcom G4 Platinum CGMS with a MARD value of 13% (10). The study on Dexcom G5 Platinum CGMS indicated a MARD value of 9.5% (11). In addition, a separate study of Dexcom G6 Platinum CGMS showed a MARD value of 9.0% (12). The Guardian Connect CGMS had a MARD value of 9.7% (13). Notably, due to limited available data within the hypoglycemic range, the accuracy of the sensors in the low blood glucose range (< 3.9 mmol/L) could not be effectively assessed. Previous studies have indicated that MARD values during hypoglycemia were significantly higher than those within the normal glucose range (14). Therefore, the focus of rtCGM in predicting hypoglycemia should be increased in the future.

The accuracy of Glunovo^®^ was impaired during rapid changes in blood glucose, especially when the blood glucose change rate surpasses 0.11 mmol/L/min. Similarly, in a study of CGM in patients with type 1 diabetes, overall MARD during acute exercise was 29.8% (15). Since CGM does not directly measure glucose concentration in the veins, its values are determined by the rate of glucose diffusion from the plasma to the interstitial fluid and the rate of glucose uptake by cells in subcutaneous tissue (5). The rate of change in glucose concentration in interstitial fluid within tissues is typically slower than that in plasma, often resulting in a delay (16). When blood glucose undergoes rapid fluctuations, this delay was amplified, which could compromise the accuracy of CGM.

The Clarke Error Grid Analysis estimated high clinical performance, with 99.61% of samples in the clinically acceptable error zones A and B. In a multicenter study focusing on the Eversense implantable CGM sensor, the results showed that 99.2% of samples were within the clinically acceptable error zones A (84.3%) and B (14.9%) (17). Moreover, real-time continuous glucose monitoring (rtCGM) has shown promising results in monitoring diabetes for peritoneal dialysis patients, with 99.9% of data points falling within zones A and B (18). The evidence mentioned above strongly supports the implementation of rtCGM, providing patients with viable monitoring options.

Several limitations should be considered. First, as subjects received standardized hospital treatment, results may not apply to home care. Second, the potential impact of confounding factors, such as patient medication profiles and the severity of diabetes, may not have been comprehensively addressed. Third, limited hypoglycemia data may impact the assessment of monitoring effectiveness in low glucose conditions. Future studies should aim for larger sample sizes to detect differences in the low blood glucose range, thereby providing more insights for physicians.

## Conclusion

In conclusion, our study highlights the enhanced accuracy of Glunovo^®^ in blood glucose monitoring for hospitalized patients, providing an alternative for diabetes assessment and management. Nevertheless, the reliability of Glunovo^®^ in low blood glucose monitoring requires verification. Further research is warranted to provide insights for the utilization of Glunovo^®^ in the future.

## Data availability statement

The original contributions presented in the study are included in the article/supplementary material. Further inquiries can be directed to the corresponding authors.

## Ethics statement

The studies involving humans were approved by Ethics Committee of Nanjing First Hospital. The studies were conducted in accordance with the local legislation and institutional requirements. Written informed consent for participation was obtained from the participants or the participants’ legal guardians/next of kin.

## Author contributions

SG: Data curation, Methodology, Software, Visualization, Writing – original draft, Writing – review & editing. HZ: Writing – review & editing, Investigation, Software, Validation. JW: Investigation, Software, Validation, Writing – review & editing. HL: Data curation, Investigation, Writing – review & editing. XS: Data curation, Investigation, Writing – review & editing. DD: Project administration, Supervision, Writing – review & editing. JM: Conceptualization, Data curation, Funding acquisition, Methodology, Project administration, Supervision, Validation, Writing – review & editing.
