# Corrigendum: Accuracy of a novel real-time continuous glucose monitoring system: a prospective self-controlled study in thirty hospitalized patients with type 2 diabetes

**DOI:** 10.3389/fendo.2024.1505073

**Published:** 2024-10-17

**Authors:** Shenghui Ge, Hui Zhang, Jun Wang, Huiqin Li, Xiaofei Su, Dafa Ding, Jianhua Ma

**Affiliations:** ^1^ Department of Endocrinology, Nanjing First Hospital, Nanjing Medical University, Nanjing, China; ^2^ Department of Endocrinology, The Second Affiliated Hospital of Nanjing Medical University, Nanjing, China; ^3^ Department of Health Management Center, Nanjing First Hospital, Nanjing Medical University, Nanjing, China

**Keywords:** Glunovo^®^, rtCGMS, type 2 diabetes, flash glucose monitoring, venous blood glucose

In the published article, there was an error in the affiliation of Hui Zhang. Instead of “Department of Endocrine, Nanjing Tongren Hospital, School of Medicine, Southeast University, Nanjing, China”, it should be “Department of Endocrinology, The Second Affiliated Hospital of Nanjing Medical University, Nanjing, China”.

The authors apologize for this error and state that this does not change the scientific conclusions of the article in any way. The original article has been updated.

